# Aripiprazole Selectively Reduces Motor Tics in a Young Animal Model for Tourette’s Syndrome and Comorbid Attention Deficit and Hyperactivity Disorder

**DOI:** 10.3389/fneur.2018.00059

**Published:** 2018-02-13

**Authors:** Francesca Rizzo, Ester Nespoli, Alireza Abaei, Izhar Bar-Gad, Dinesh K. Deelchand, Jörg Fegert, Volker Rasche, Bastian Hengerer, Tobias M. Boeckers

**Affiliations:** ^1^Department for Child and Adolescent Psychiatry and Psychotherapy, Ulm University, Ulm, Germany; ^2^Institute of Anatomy and Cell Biology, Ulm University, Ulm, Germany; ^3^Boehringer Ingelheim Pharma GmbH & Co. KG, CNS Diseases, Biberach an der Riss, Germany; ^4^Core Facility Small Animal Imaging, Ulm University, Ulm, Germany; ^5^Department of Internal Medicine II, Ulm University Medical Center, Ulm, Germany; ^6^Leslie and Susan Gonda (Goldschmied) Multidisciplinary Brain Research Center, Bar-Ilan University, Ramat Gan, Israel; ^7^Center for Magnetic Resonance Research, University of Minnesota, Minneapolis, MN, United States

**Keywords:** adolescence, spontaneously hypertensive rat, 1H magnetic resonance spectroscopy, attention-deficit/hyperactivity disorder, Tourette’s syndrome, striatum, metabolite absolute concentration, stereotypies

## Abstract

Tourette’s syndrome (TS) is a neurodevelopmental disorder characterized primarily by motor and vocal tics. Comorbidities such as attention deficit and hyperactivity disorder (ADHD) are observed in over 50% of TS patients. We applied aripiprazole in a juvenile rat model that displays motor tics and hyperactivity. We additionally assessed the amount of ultrasonic vocalizations (USVs) as an indicator for the presence of vocal tics and evaluated the changes in the striatal neurometabolism using *in vivo* proton magnetic resonance spectroscopy (1H-MRS) at 11.7T. Thirty-one juvenile spontaneously hypertensive rats (SHRs) underwent bicuculline striatal microinjection and treatment with either aripiprazole or vehicle. Control groups were sham operated and sham injected. Behavior, USVs, and striatal neurochemical profile were analyzed at early, middle, and late adolescence (postnatal days 35 to 50). Bicuculline microinjections in the dorsolateral striatum induced motor tics in SHR juvenile rats. Acute aripiprazole administration selectively reduced both tic frequency and latency, whereas stereotypies, USVs, and hyperactivity remained unaltered. The striatal neurochemical profile was only moderately altered after tic-induction and was not affected by systemic drug treatment. When applied to a young rat model that provides high degrees of construct, face, and predictive validity for TS and comorbid ADHD, aripiprazole selectively reduces motor tics, revealing that tics and stereotypies are distinct phenomena in line with clinical treatment of patients. Finally, our 1H-MRS results suggest a critical revision of the striatal role in the hypothesized cortico-striatal dysregulation in TS pathophysiology.

## Introduction

Tourette’s syndrome (TS) is a neurodevelopmental tic disorder occurring before the age of 18 and is characterized by motor and vocal tics that wax and wane over time (DSM-V). Tics in TS usually follow a remitting course, improving symptomatology in late adolescence or early adulthood. Attention-deficit/hyperactivity disorder (ADHD) is a neurodevelopmental disorder characterized by a persistent pattern of inattention and/or hyperactivity-impulsivity occurring before the age of 12 that interferes with overall functioning and/or development (DSM-V). In almost half of TS cases, the two pathologies co-occur in children (1). Comorbid ADHD commonly antecedes first tics and, during TS natural course was shown to be more impairing on patients’ quality of life then the tics themselves (2–5). This emphasizes the need for comprehensive *in vivo* approaches for studying TS, i.e., animal models able to resemble TS comorbidities together with the tic-like phenotype. A tic is a sudden, rapid, recurrent, non-rhythmic motor movement, or vocalization that can vary in frequency, intensity, duration, and anatomical localization. Tics are typically preceded by an uncomfortable phenomenon called “premonitory urge” and can be voluntarily suppressed by most patients for a short period of time (DSM-5). In patients, tics and stereotypies coexist and can be easily confused, leading to a delayed TS diagnosis or to a wrong treatment approach (6). In animal models, stereotypies belong to the spontaneous repetitive movement repertoire and their overexpression (hyper-grooming/-sniffing/-climbing) following genetic or pharmacological manipulation has been used as tic-like phenotype in modeling TS (7–10). A phenotype characterized by bousts of repeated and somatotopically organized movements have also been reached in different species applying bicuculline directly to the dorsolateral striatal region (11–14).

At the moment, a lack of homogeneity in the terms employed to identify motor phenomena in animal models of TS exists, mostly due to the variety of approaches used to achieve the ticcing phenotype.

In this study, we aim to overcome some of the actual TS model limitation choosing the striatal disinhibition paradigm applied first to a small cohort of Wistar Kyoto (WKY) rats and then to young spontaneous hypertensive rats (SHRs).

Among the existing variety of ADHD animal models, SHRs represent the most commonly used model showing all of the core disorder’s symptoms (inattention, impulsiveness, and aggressiveness) even though its predictive validity remains limited (15, 16). However, SHRs demonstrating tic-like movements as well as tic-expressing animals showing comorbid ADHD symptoms have not been documented so far. Validity for the single features were reported: hyperactivity was associated with specific bicuculline injections sites in the dorsal striatum and dorsal GPe of primates (12, 17) or in the ventral striatum of the adult rat (13); and attention deficit occurred after injections in associative regions of the GPe (17). In our model, the unilateral bicuculline injection within the anterior dorsolateral striatum resulted in a juvenile spontaneously hyperactive animal model that displayed transient tics, recapitulating the core-symptoms of both TS and comorbid ADHD. The phenotype was stable during the entire adolescence, a period of time that in the rat is placed between postnatal days (PNDs) 35 and 50 (18).

To test the predictive validity of our model, we tested aripiprazole, which was recently shown to effectively modulate the total tic score (45.9 and 54.2% with low and high dose, respectively) in a large multinational study (19). Due to its better-tolerated side effect profile, aripiprazole has been introduced as first-line treatment for tic management, overcoming the use of other typical antipsychotics.

In our model, aripiprazole dramatically reduced the number of tics and their latency immediately after the first administration and during consequent administrations. Over the same timespan hyperactivity, stereotypies and spontaneous USVs did not change their pattern following neither focal bicuculline injection (leading to tic generation) nor systemic aripiprazole application (leading to tic reduction), suggesting a unique neurological pathway for tic generation that is separate from those related to the other motor or vocal comorbid phenomena.

Finally, we used our prior established proton magnetic resonance spectroscopy (1H-MRS) protocol to investigate changes in the neurochemical composition of the bilateral striatum with the intention to identify a neurochemical signature, if present, of the “just occurred” bicuculline-induced tic as well as of the effective action of the aripiprazole treatment (20). Surprisingly, our results revealed an “unaltered striatum,” i.e., only few changes occurred following the tic session or of the effective drug treatment, raising questions on the role of the striatum within the supposed dysfunctional cortical–striatal–thalamic–cortico circuitry in TS.

## Materials and Methods

### Animals

Thirty juvenile male SHRs (Charles River Laboratories, Sulzfeld, Germany) were used in this study. Up to four pups per cage were housed together with their mother until PND 21. For the entire duration of the experiment, the rats had free access to standard laboratory rodent chow and water, and were housed in a room with 12-h light-dark cycle. Temperature and humidity were maintained at 22 ± 1°C and 45–55%, respectively. All the experiments were approved by the Committee for Animal Experimentation of the University of Ulm and the regional administrative authority in Tübingen (TVA No. 1200).

### Study Design

The study design is to analyze behavioral and neurochemical alterations following bicuculline/saline focal injection and drug/vehicle systemic acute administration in a longitudinal manner during the period of adolescence evaluating PND 35, 42, 46, and 50.

### Experimental Groups

**Figure 1:**
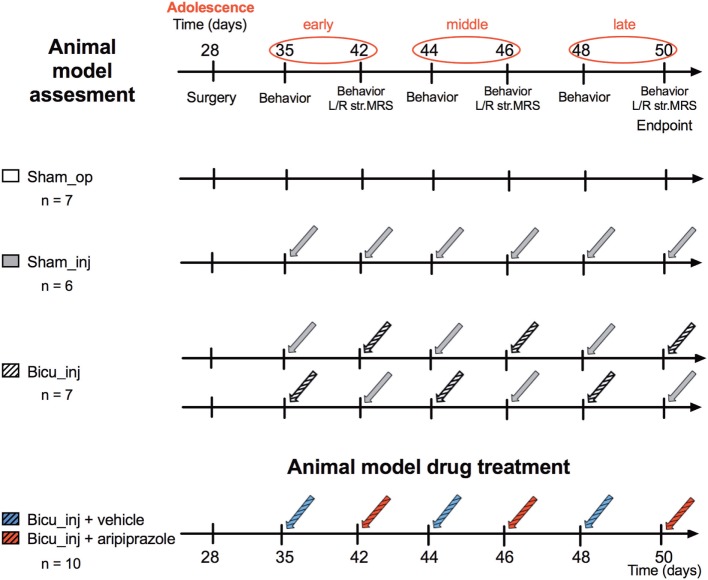
Experimental design. Design of the study for the animal model assessment (top) and the drug treatment (bottom).

The animals were randomly divided to four different groups: the “Sham_op” group (*n* = 7) in which the animals underwent the cannula implantation procedure only; the “Sham_inj” (*n* = 6) group in which cannulated rats received saline intracerebral microinjection; the “Bicu_inj” group (*n* = 7) in which cannulated rats received saline/bicuculline intracerebral microinjection; and the “Bicu_inj + vehicle/aripiprazole” (*n* = 10) in which cannulated rats alternatively received the acute i.p. treatment with vehicle or aripiprazole. The experimental scheme is shown in Figure 1.

### Experimental Procedures

**Figure 2:**
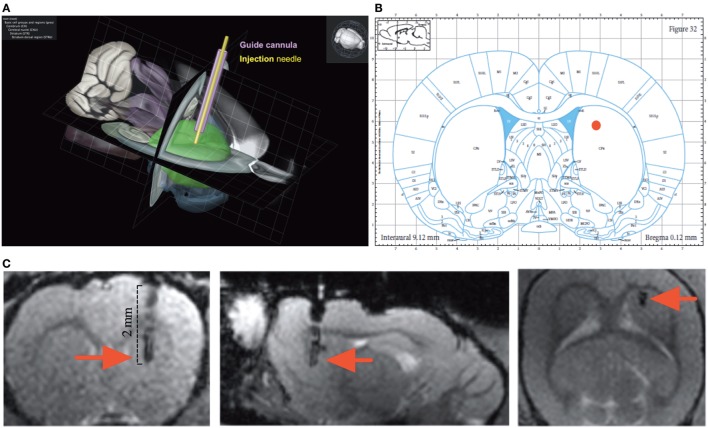
Anatomy. **(A)** Tridimensional reconstruction of the cannula implantation (purple) and the injection needle (yellow) site within the rat dorsolateral striatum (green) (adapted from http://brain-map.org). **(B)** The site of injection (red dot) overlaid on the Paxinos and Watson rat brain atlas. **(C)** Injection site at the end of the cannula position within the sagittal, coronal, and axial anatomical scan of the rat brain (red arrow).

The surgery and microinjection protocols as well as the USV recording methods are detailed in Supplementary Material. The targeted brain coordinates chosen for this study were the result of our collaboration with EN at Boehringer Ingelheim: (AP: +0.15, ML: +0.25, DV: +0.45) (Figures 2A–C).

### Drug Treatment

In this vehicle-controlled designed study, the acute pharmacological intervention started on PND 35, when the rats were weighing 100 ± 30 g, and ended on PND 50 when the rats were weighing 180 ± 10 g. Drug preparation, dosage, and administration method of the aripiprazole (*t*_1/2_ 10.9–19.4 days in whole blood and 10.0–14.7 days in plasma *in vivo*, 31 min *in vitro*) or vehicle treatment was described in details elsewhere (20).

### Behavioral Assessment

#### Stereotypies Analysis and Tic Quantification

High-speed (50 fps) video streams were used for stereotypic behavior quantification using The Observer 13 (Noldus). Within the same 5 min of tracked locomotion, stereotypies like grooming (behavioral chain composed of paw-licking, paw-rubbing over the head, licking and rubbing the side of the body, the ano-genital region, and the tail), rearing (vertical locomotion up or down a vertical surface using the forepaws only), and climbing behavior (vertical locomotion up on a vertical surface using both forepaws or pull and hind paws or push to grasp footholds and haul themselves up) were analyzed in terms of frequency (counts) and total time spent (seconds) performing the specific behavior. Frame-by-frame analyses were also used for detailed temporal analyses of the tics. The timing of the first frame in which the tic-related deflection of the relevant body part was observed was defined as tic latency time. Tic quantification in this study is referred to a deflection of the right forelimb as common denominator among other features of the bicuculline-induced phenotype (13).

#### Spontaneous Locomotor Activity in Standard Cage Type-III Analysis

All behavioral experiments were conducted during each of the six timepoints and immediately before anesthesia induction on the MRS session days. For simplicity, in the graphs we grouped the six timepoints (PNDs 35, 42, 44, 46, 48, and 50) to three periods referring to them as early-, mid-, and late-adolescence. On each test day, animals were weighed before testing and placed in the center the individual test cage (Plexiglas standard cage type III, 425 mm × 266 mm × 155 mm) to record locomotor activity. Each video was analyzed using the Ethovision 10.XT software (Noldus) for animal behavior quantification. Horizontal locomotor activity was tracked during 5 out of 10 videotaped minutes (from minutes 2 to 7 of each session) and was analyzed to extract the mean distance moved (cm), mean velocity (cm/s), and the ratio of the time spent moving vs. not moving.

#### Proton Magnetic Resonance Spectroscopy (1H-MRS) Session

Magnetic resonance spectroscopy assessment was conducted using the same equipment and spectra acquisition protocol and exclusion criteria as previously described (20). MRS was carried out on a 11.7T animal scanner (Bruker BioSpin, Ettlingen, Germany) equipped with a volume resonator for excitation and a phased-array surface coil for signal reception. Two regions of interest were targeted with 18.5-µL total voxel volume, respectively, positioned in the left and right striatum.

Local magnetic field homogeneity was optimized and MRS was performed with a short-echo-time STEAM (STimulated Echo Acquisition Mode) (21) sequence at TR/TE/TM: 5000/3.5/10 ms, 5-kHz spectral width, 2,048 data points, and 256 averages. Water-suppressed spectra were collected over a total acquisition time of 21 min. Absolute metabolite concentration in mM, i.e., mmoL/L VOI, was estimated for all the metabolites by LCModel (Version 6.3-1C, S. Provencher, Oakville, ON, Canada), a user-independent fitting routine on a basis set provided by Provencher and CMRR-written MATLAB code. The unsuppressed water signal was used as an internal reference (22, 23). All spectra were visually inspected for the presence of spurious resonances or artifacts prior to the statistical analysis. Cramér –Rao lower bounds (CRLBs) of LCModel analysis were used to assess accuracy and reliability of the fitting, which is regarded as the metabolites concentration estimated errors and reflect the estimated %SD of the metabolite fit (24, 25). CRLB >50% was considered as exclusion criterion for metabolite evaluation. Other rejection criteria, such as poor SNR (<6), existence of strong baseline distortions, and line widths [full-width at half-maximum peak height (FWHH)], which exceeded the expectations limits, were applied (26).

Magnetic resonance spectroscopy investigation immediately followed behavioral recordings. Spectra were acquired longitudinally from the same animal on PNDs 42, 46, and 50 under anesthesia (0.8–1.8% isoflurane in 1:5 oxygen: air mixture supplied by face mask). Consistent with published recommendations for prolonged anesthesia (27) exposure, respiration rate was monitored throughout the scanning and isoflurane was adjusted to maintain respiration within specified target range (35–45 rpm). Body temperature was maintained using a heated hydro system of tubes constantly supplying the animal holder with warm water. The total duration of MRS including animal preparation was 90–110 min. Depending on the timepoint of the experiment, after completion of the MRS measurements animals were either brought back to their cages or sacrificed by decapitation. The brain was dissected and stored −80°C for further *ex vivo* analysis.

### Statistical Analysis

Data are reported as mean ± SEM unless otherwise stated. Behavioral data analysis was done using Mann–Whitney *U* test. MRS data analysis of treatment-related changes in neurometabolite absolute concentrations were assessed separately for each metabolite by one-way ANOVAs. The presence of variance heterogeneity was excluded by the Levene test. Data were tested for normal distribution and Bonferroni *post hoc* tests were applied to account for multiple testing arising from pairwise comparisons. Specifically, each timepoint of the drug-treated groups was compared with the respective vehicle-treated group within the same timepoint. All differences were considered significant for *p* < 0.05. The analyses were conducted using GraphPad Prism 7.

## Results

### Motor Tics and Effect of the Drug Treatment

Unilateral bicuculline intrastriatal microinjections evoked tic-like movements, while no tics were observed in the Sham_op and Sham_inj groups. Tics always appeared in the contralateral side to the injection and none were observed in the ipsilateral side (Video S1 in Supplementary Material). Tics began with a brief jaw tremor and eyeblink and were followed by tics in the forepaw that ranged over time from small twitches to strong deflections of an entire limb or even the torso. During the course of a single session, small tics would appear rapidly increasing their amplitude and frequency, maintaining a stereotypic feature throughout the session until they gradually decreased and reached complete cessation similar to the sessions described in Bronfeld et al. (13). Forelimb and facial tics were expressed alone or in combination following the bicuculline microinjection in the anterior striatum. Aripiprazole treatment was administered 30 min prior to bicuculline injection in order to meet the highest point of the pharmacokinetic curve of aripiprazole.

**Figure 3:**
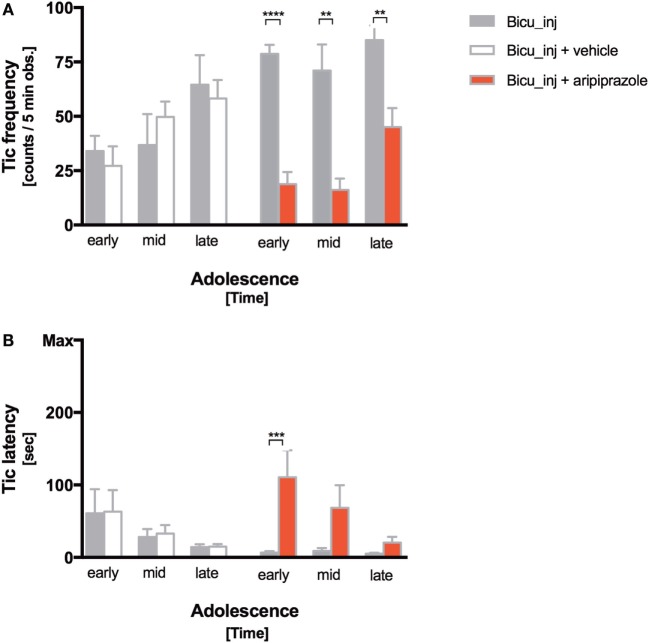
Tic expression following aripiprazole. **(A,B)** Frequency (tic counts/5-min observation) **(A)** and latency (time to first tic) **(B)** of bicuculline-induced motor tics at early, mid- and late adolescence. Stars indicate levels of significance (*P < 0.05, **P < 0.01, ***P < 0.001, and ****P < 0.0001). White, red, and gray columns indicate vehicle treatment, aripiprazole treatment, and bicuculline injected only groups, respectively.

Tic frequency decreased significantly after aripiprazole acute administration at all the timepoints during adolescence (early = *p* < 0.0001; mid- and late *p* < 0.005), while differences between the bicuculline-injected rats treated with vehicle did not show significant alteration compared with the non-treated group (Figure 3A). Aripiprazole also led to a delayed tics onset that was the highest at the earliest timepoint (*p* < 0.0005) (Figure 3B). Tic latency was 1.5 min ± 30 s and became shorter over time following subsequent bicuculline microinjections (Figure 3B). During the 5-min observation time, the average tic frequency was 55.15 ± 23.49 tics corresponding to a tic rate of 11.03 ± 5.04 tics/min.

### Spontaneous Locomotion

Locomotion was assessed to detect changes of spontaneous locomotor activity due to either the intracerebral (saline/bicuculline) or intraperitoneal (vehicle/aripiprazole) injections. The presence of the cannula as well as the saline injection did not affect the spontaneous locomotion over time (total distance moved and velocity) and neither did intrastriatal injections of bicuculline and aripiprazole acute treatment with the only exception of PND 48 (Figure S2 in Supplementary Material).

### Stereotypies Quantification

Stereotypic movements were investigated in order to assess the intracerebral injection effect on the spontaneous stereotypical repertoire among all four cohorts.

Significant differences in rising, grooming, and climbing frequency were not detected in any cohort (Figure S2 in Supplementary Material). Stereotypes differences in latency and total duration are randomly present over the late-adolescence among the groups, showing an overall non-significant impact due to injection or vehicle/drug treatment (Figure S3 in Supplementary Material).

### Spontaneous Ultrasound Vocalizations

**Figure 4:**
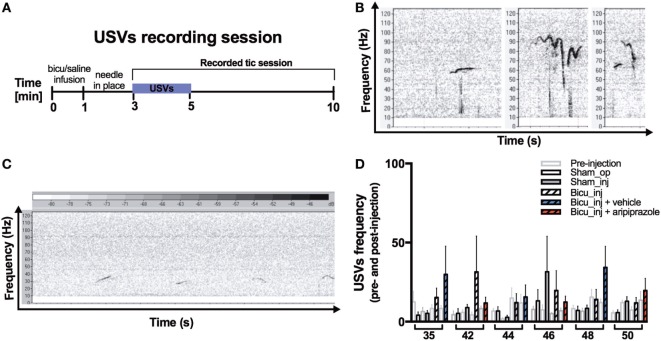
Ultrasonic vocalizations. **(A)** Experimental design of the ultrasonic vocalization (USV) recording session. **(B)** Flat (left), frequency-modulated (FM) (center), and two-frequency step (FS) (right) calls representative of the whole range of the rat ultrasonic vocalization. **(C)** Representative juvenile rat spontaneous USV spectrum recorded 3 min after bicuculline injection. Calls appear flat, short, and rhythmically repeated **(D)** Graph showing USV frequency pre-and post-bicuculline injection.

We investigated for the first time the spontaneous ultrasound vocalizations of the bicuculline injected young rodent model and control groups. The USV recording session took place right after the injection with bicuculline or saline and was performed at the beginning of the behavioral recording session (Figure 4A). Qualitative analysis showed that rats belonging to all the groups emitted the whole range of flat (resembling a flat line, i.e., no more than one change in frequency direction not exceeding 5 kHz), frequency-modulated (FM) (with a minimum of two waves, with more than 5-kHz frequency difference within a wave), and two frequency step (FS) (complex USVs with a sudden jump in frequency of more than 5 kHz) (Figure 4B). Qualitatively, USVs appeared mostly short, flat, and rhythmically repeated in those rats that received the bicuculline injection (Figure 4C). Quantitative analysis of USV frequency both before and after bicuculline injection did not reveal any significant difference compared with those belonging to the sham-operated and sham-injected groups. Finally, aripiprazole treatment did not influence the USV frequency as compared with the vehicle treatment effect (Figure 4D).

### *In Vivo* (1H) MRS Absolute Quantification of Striatal Neurometabolites

**Figure 5:**
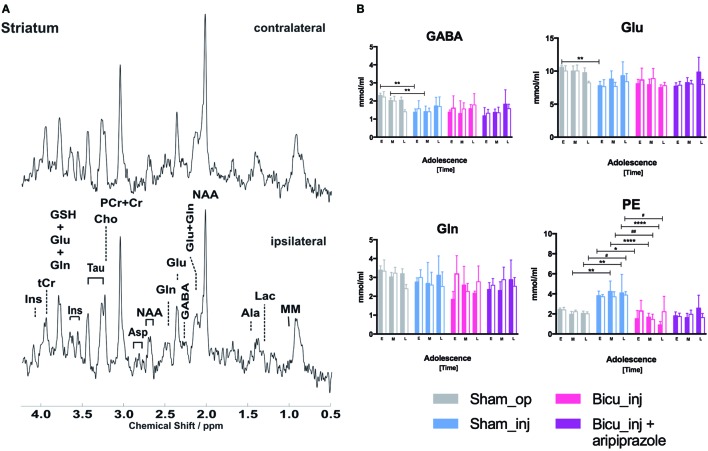
Proton magnetic resonance spectroscopy (MRS). **(A)** Representative spectra obtained using multislice FLASH, TR/TE = 191/5 ms, 17.5° flip angle, 27-mm field of view, and 0.75-mm slice thickness from voxel localized within both the ipsilateral and contralateral site of injection. **(B)** Modulation of γ-aminobutyric acid (GABA), glutamate (Glu), glutamine (Gln), and phosphatidylethanolamine (PE) as detected by *in vivo* 1H-NMR MRS in the right striatum of sham operated (gray columns), sham injected (blue columns), bicuculline injected (pink columns), bicuculline injected and aripiprazole treated (purple columns), and the corresponding contralateral side (white filled columns). Ipsilateral (*) and contralateral (#) different significant levels are indicated for each timepoint (E: early, M: middle, and L: late) of adolescence: *p < 0.05, **p < 0.01, ***p < 0.001, and ****p < 0.0001.

Representative spectra derived from voxels centered in the striatum localized next to the cannula in the ipsilateral hemisphere as well as in the contralateral side are shown in Figure 5A. Intra-group analysis did not show significant differences of any neurometabolite levels between the left and right striatal hemispheres (colored column vs. adjacent white column, Figure 4; Figure S5 in Supplementary Material) in rats belonging to the same groups for all of the cohorts. Inter-group comparison of the left and right striatum among the cohorts shows variations in phosphatidylethanolamine (PE) striatal levels that significantly increase in the cannula side during mid- and late-adolescence (*p* < 0.0005) in the Sham_op group compared with the Sham_inj group and dramatically decreases at any timepoint (early *p* < 0.05; mid- and late *p* < 0.0001) when comparing the Sham_inj group with the Bicu_injected one. The same trajectory can be observed for PE also in the intact side of the striatum, especially at the latest timepoint between the Sham_op and Sham_inj groups but also at the middle (*p* < 0.005) and late ones (*p* < 0.05) between the Sham_inj and Bicu_inj rats. Comparisons among the rest of the cohort reveal few significant changes in neurometabolite level among which a bilateral decrease of GABA (*p* < 0.05) at the latest timepoint can be addressed to the systemic treatment with aripiprazole when comparing the Bicu_inj + ari group and its own control—the Bicu_inj group (Figure 5B).

## Discussion

Data from human imaging studies, as well as electrophysiological and postmortem anatomical studies (28–31) have contributed to our understanding of the pathophysiology of TS. Dataset obtained over the last decades from *in vivo* studies investigating the underlying pathology are heterogeneous and largely dependent on the ample variety of TS animal models available (5). In this study, we introduce a novel animal model that combines core features of TS with important comorbidity traits. The tic session was similar in the hyperactive/hypertensive and normotensive cohorts (Video S1 in Supplementary Material). Strain-related differences could be qualitatively noticed: in SHRs tic latency was reduced compared with WKY and tics appeared to reach faster a high intensity, giving to SHR tic session a “prone to tic” shape. SHRs are known to have a basal dopamine efflux within the striatum that is significantly higher than in long evans (LE) (32), Sprague–Dawley (SD) (33), and WKY (34). Dopamine is reported to directly inhibit GABA_A_ receptors that are immediately adjacent to dopamine release sites in the striatum (35). The existing higher basal efflux of dopamine in the SHR strain (32–34) may enhance the disinhibition exerted by bicuculline injection resulting in a stronger and slightly shorter tic session compared with the WKY, as we observed in our experiments. Tic latency and the total tic-session duration were shorter compared with the SD and LE adult model, but might arise due to the difference in age, strain, and sex.

Despite the invasive nature of the surgery and experiments, the procedures used to achieve the tic phenotype in our model did not affect the whole body and brain weight (Figure S1 in Supplementary Material) throughout the duration of the experiments. The procedures also did not alter the typical hyperactivity of the SHR strain (Figure S2 in Supplementary Material), maintaining the ADHD-comorbid characteristic. Bicuculline injection exerted tic movements but did not influence the frequency or latency of any other behavior within the stereotypic repertoire (Figures S3 and S4 in Supplementary Material). This establishes a clear-cut division between the neurological mechanism responsible for the tic generation and the one responsible for stereotypies.

Underpinning the circuit underlying such a result was not the focus of this study, but it connects with the call for standardization of the preclinical model for TS due to the still remaining confusion between these two motor phenomena (tics and stereotypies) often used in the tic-model literature with an interchangeable meaning (36). Tic-like movements in animal models are often confused with stereotypies, though they are separated clinical entities. Especially in young TS patients, however, comorbidities like ADHD are also commonly seen.

We tested aripiprazole acute treatment efficacy on our model. Aripiprazole reduced by threefold bicuculline induced tics immediately after the first administration and this effect persisted over consequent treatments (Figure 3A). Aripiprazole efficacy can also be appreciated through the reduced latency of tics (Figure 3B), even though its powerful effect at early timepoint is reduced toward the end of the adolescent period. Aripiprazole significantly ameliorated tics, but did not exert any notable effect on the frequency or latency of repetitive behaviors or on the numbers of USVs, as well as on the hyperactivity. This evidence strengthens our hypothesis that these three features, even though all existing in TS clinical presentation, at least in rats belong to different neuronal circuits. In a non-human primate proposed-model for TS, distinct but inter-digitated neuronal circuits of the basal ganglia were associated with different symptoms. In particular, tic-like movements were associated with the medial and lateral premotor cortex and sensorimotor parts of the basal ganglia; hyperactivity was associated with the prefrontal dorsolateral cortex; and associative territories of the basal ganglia and stereotyped behaviors were linked to the orbitofrontal cortex and limbic part of the basal ganglia (17, 37, 38).

Our results showed for the first time that repeated bicuculline injections, despite its invasiveness, did not compromise the ability of our rats to emit the whole range of spontaneous USVs (Figure 4B). When comparing the number of calls pre- and post-bicuculline injection, our results did not demonstrate statistically significant changes in USVs. There is evidence that nucleus accumbens would have been an alternative site of injection putative for evoking vocal tics as demonstrated in non-human primates (39).

We also performed for the first time *in vivo* 1H-MRS longitudinally in the rat model of striatal disinhibition with the aim of detecting neurochemical changes within the striatum that could be connected with the observed bicuculline-induced phenotype. Unexpectedly, comparing both hemispheres within each cohort, no significant differences were detected among the pool of investigated metabolites. When differentially analyzing the same brain hemisphere among the different cohorts, only PE resulted in a significant increase after saline injection and its levels dramatically dropped after each bicuculline injection. The same phenomenon, even though to a smaller extent, was observed in the contralateral intact side of the brain. PE content in the mammalian brain is 45% of the total phospholipids and serves multiple important cellular functions. It is a precursor of anandamide, the ligand for cannabinoid receptors in the brain (40). The dramatically decreased level of PE observed in our study after bicuculline injection could actively contribute to the tic phonotype. A study using an indirect cannabinoid agonist that enhances endogenous anandamide levels was conducted on another proposed animal model of TS, the DOI-induced head twitch responses mice in four different strains resulting in ameliorating of the tics without suppressing other behaviors (41). Among the variety of TS etiological theories available (31, 42), a pathophysiological role for cannabinoids in TS was also hypothesized following the observation of patients who reported that prior use of marijuana led to a reduction or complete remission of motor and vocal tics as well as of other comorbid symptoms (43).

On the neurochemical level, we could not detect any significant change within the analyzed metabolites as a readout of the aripiprazole treatment. In our previous work, we were able to detect even minimal changes of the neurochemicals in the intact brain of WKY and SHR under subchronic aripiprazole treatment condition using the same dosage and administration route (20). A potential hypothesis is that the effect of the injection procedure itself produced a stressful impact significantly bigger than the one exerted by the administered neuroactive compounds (bicuculline, aripiprazole). Thus, if a neurochemical effect is present, it probably lies below the detectable cutoff of our established spectroscopy protocol. More importantly, an interesting explanation for the “unaltered striatum” we revealed, despite an obvious bicuculline-induced phenotype and its modulation using aripiprazole, could be that the striatum itself is able *per se* to cope with local chemical/mechanical alterations. This would result in a protection of its neurometabolites homeostasis, maintaining a preserved functional output to the cortex that, at least in our model, is hypothesized to be dysfunctional but that needs to be further investigated. A recent support for this direction has been given by a study in which TS participants showed distributed differences in the activation of multiple cortical regions with differences in functional reactivity and that may account for heterogeneity in the symptomatic expression of TS and its comorbidities (44).

The lack of standardization procedures and the absence of a rigorous evaluation tool led us to stick to the traditional idea of testing and discussing the three aspects of model validity: construct (the rationale behind the model matches the pathological hypothesis), face (the model shows symptoms similar to the patients’ ones), and predictive (the model and patients response to the same treatment is similar) (45–47). In terms of construct validity, the rat model of striatal disinhibition achieved *via* bicuculline injection fits Mink’s tic generation hypothesis. The loss of inhibition within a specific neuronal population of the striatum results in the inability to inhibit a movement that should be prevented (48). Such a loss is experimentally induced in the rat by the GABA_A_ antagonist bicuculline intrastriatal injection that evokes a motor phenotype characterized by bouts of movements that fluctuate in frequency and duration over time, resulting in a tic session comparable with some extents to those experienced by TS patients (13). Construct and face validity, both reasonably achieved in this model, convinced us to use this paradigm for testing aripiprazole efficacy in tic management (predictive validity).

This study is the first of its kind and potentially a starting point for future proliferation of non-invasive *in vivo* spectroscopic brain investigations using our 1H-MRS protocol and the newly proposed animal model for TS and comorbid ADHD. We are aware that the main limitation of this study, the “acute” nature of the tic session, has been recently overcome by a chronic tic model achieved so far only in the adult female LE rats (49). Our belief is that such a method would perfectly fit our TS-ADHD model at young age and would be feasible to our 1H-MRS protocol. Focusing on different regions of interest, we are optimistic that such a study-design in the future would reveal the neurochemical signature of a tic and/or its pharmacological treatment, adding striking new information to prior TS pathophysiological hypothesis (49–52).

## Conclusion

To the best of our knowledge, this is the first animal model able to provide high degrees of construct, face, and predictive validity as a model for TS and comorbid ADHD. We believe that our model together with our 1H-MRS protocol represents a valid tool for TS pathophysiological investigation as well as for testing novel treatment approaches.

## Ethics Statement

All the experiments were approved by the Committee for Animal Experimentation of the University of Ulm and the regional administrative authority in Tübingen (TVA No. 1200).

## Author Contributions

FR was primarily involved in the writing of the ethical allowance and execution of the research project, as well as the design and execution of the statistical analysis and the writing of the manuscript. EN contribution was fundamental in the establishment of the surgery and behavioral protocols. DD and AA established the 1H-MRS *in vivo* protocol, AA was responsible for the spectra quality control and data screening. IBG expertise was essential for the research project rationale, experimental design and the critical review of the manuscript from the preclinical point of view. VR and JF critically reviewed the manuscript contributing to the clinical importance of MRI in psychiatric diseases and to the bench-to-bed importance of this study respectively. BH supported the study and helped in the experimental drug testing design. TMB conceived, supported and organized the research project and critically contributed to the writing and reviewing process of the manuscript.

## Conflict of Interest Statement

During the last 5 years Professor JF received research funding from the EU, DFG (German Research Foundation), BMG (Federal Ministry of Health), BMBF (Federal Ministry of Education and Research), BMFSFJ (Federal Ministry of Family, Senior Citizens, Women and Youth), German Armed Forces, several state ministries of social affairs, State Foundation Baden-Württemberg, Volkswagen Foundation, European Academy, Pontifical Gregorian University, RAZ, CJD, Caritas, and Diocese of Rottenburg-Stuttgart. Moreover, he received travel grants, honoraria, and sponsoring for conferences and medical educational purposes from DFG, AACAP, NIMH/NIH, EU, Pro Helvetia, Shire, several universities, professional associations, political foundations, and German federal and state ministries. Clinical trials for Lundbeck, BMBF, and Servier. Professor Fegert holds no stocks of pharmaceutical companies. The remaining authors declare no conflict of interest.

## Supplementary Material

Video S1 Typical tic session after bicuculine injection in SHR and WKY rats.

## Abbreviations

ADHD, attention-deficit/hyperactivity disorder; Ala, alanine; Asp, aspartate; DOI, (±)-2,5-dimethoxy-4-iodoamphetamine; GABA, γ-aminobutyric acid; Glu, glutamate; Gln, glutamine; GSH, glutathione; Ins, inositoles; LE, long evans; MRS, magnetic resonance spectroscopy; OCD, obsessive compulsive disorder; PE, phosphatidylethanolamine; PPI, pre-pulse inhibition; SD, Sprague–Dawley; SHR, spontaneously hypertensive rat; Tau, taurine; tot. Cho, total choline; tot. Cr, total creatine; tot. NAA, total *N*-acetylaspartate; TS, Tourette’s syndrome; USVs, ultrasonic vocalizations; WKY, Wistar Kyoto.
