# Supplementary material for: Aripiprazole Selectively Reduces Motor Tics in a Young Animal Model for Tourette’s Syndrome and Comorbid Attention Deficit and Hyperactivity Disorder
Source: Front Neurol. 2018 Feb 13;9:59. doi: 10.3389/fneur.2018.00059 (PMC5816975; doi:10.3389/fneur.2018.00059)
Supplement: Supplementary file 2 [file Data_Sheet_1.PDF]

Supplementary Material and methods

Surgical procedure

Rats were sedated with 5% isoflurane and anesthesia was maintained using a mixture of isoflurane 0.5–1% and oxygen. The rat's head was fixed in a stereotaxic frame (Stoelting Co., Wood Dale, IL, USA). After sterilization of the skin and local infiltration with lidocaine (10 mg/ml) to reduce pain, an incision was made in order to expose the skull surface. Connective tissue was removed and the skull surface cleaned. Holes were drilled in the skull targeting the left hemisphere anterior dorsolateral striatum (AP: +0.15, ML: +0.25, DV: +0.45) (Fig.2 a,b) (Paxinos and Watson, 2007). Guide cannulae (PEEK 25 G tubes) were inserted to a location 2 mm above the injection target and sealed with a cannula-dummy (Tygon wire 28 G). The implantation was secured to the skull with 3 plastic screws and dental acrylic cement (Coltene/Whaledent Inc., Cuyahoga Falls, OH, USA). Norocarp (Carprofen, 4 mg/kg) was injected subcutaneously post-surgery to relieve pain. Experimental sessions began after the animals recovered from surgery (typically 7 days).

Microinjections

We used striatal microinjections of bicuculline, a competitive $GABA_A$ antagonist, which can also inhibit SK channels (Stocker et al., 1999) to evoke motor tics. Bicuculline methiodide (Sigma-Aldrich, Schnelldorf, Germany, https://www.sigmaaldrich.com/content/dam/sigma-aldrich/docs/Sigma/Product_Information_Sheet/b9130pis.pdf) was dissolved in physiological saline to a final concentration of 1 µg/µl. A volume of 0.5 µl was pressure injected at a constant rate of or 0.5 µl/min (NE-1000, New Era Pump Systems, Farmingdale, NY, USA) using a 10 µl syringe (Hamilton, Reno, NV, USA). The injection was applied through an injection cannula (PEEK 30 G tube), which was connected to the syringe via a flexible tube (Tygon micropore tube, Component Supply Company, Fort Meade, FL, USA). The injection cannula was manually inserted into the guide cannula which was fixed in the acrylic, with its tip located 2 mm past the tip of the guide cannula in the striatum. Microinjections were performed on each time point starting from PND 35 with saline or bicuculline depending on the experimental group. The site of injection and the cannula localizations are shown in Fig.2

Spontaneous USVs recording session

Juvenile male spontaneous vocalizations pre and post bicuculline/saline injection or no injection were analyzed in the experimental cage also used on the same six timepoint used for behavioral recording.89

Ultrasonic vocalizations were recorded by a condenser microphone (CM16; Avisoft Bioacoustic, Berlin, Germany), sensitive to frequencies of 10-150 kHz, which was mounted 20 cm above the center of the experimental cage. Ultrasonic Vocalizations were recorded with Avisoft recorder Software (UltraSoundGate 116 USB, version 3.2 Avisoft Bioacoustics, Berlin, Germany). The settings of the recorder software were set to sampling frequency of 300 kHz and a 16 bit format. For further acoustical analysis, WAV file recordings were transferred to SASLab pro Software (version 4.5, Avisoft Bioacoustics) and a Fast Fourier transform (FFT) was conducted (512 FFT length, Hamming window, time resolution: 75 % overlap. The spectrogram was generated at 586 Hz frequency resolution and a time resolution of 0.4267 ms. Background noise was eliminated by cutting of frequencies lower than 15 kHz with a high pass filter (Kemo VBF 10M, 132 db/ octave slope).

Supplementary results

Experimental procedures effects on body and brain weight.

Experimental procedures safety was checked taking note of the rats body and brain weight respectively during the whole duration and at the endpoint of the experiment. The cannula implantation performed on PND28 (rats weight 55±5 g) as well as the subsequent intrastriatal injection of saline/bicuculline and the intraperitoneal acute vehicle/aripiprazole treatment, did not affect the physiological weight gain during the entire experimental period (FigS1 a). Brain weight collected at the endpoint of the experiments did not show any significant difference among the groups (160±10 g) (FigS1 b).

FigS1: **Brain and body weight changes.** (A) Age- and treatment- condition related changes of mean (±SEM) body weight. The rats body weight increased with age during adolescence. There were no significant differences between groups tested with Student's t-test. (B) Treatment-related differences of total brain weight (±SEM) show no differences at postnatal day 50 (late adolescence). Differences between differently treated groups were not significant when tested by Student's t-test. Rats sham operated (white columns), sham injected (grey columns), bicuculline injected (Blacked striped white columns), bicuculline injected and treated with vehicle (Blacked striped blue columns) and bicuculline injected and treated with aripiprazole (Blacked striped red columns) are shown.

FigS2: **Locomotion changes.** Total distance moved (cm) (A) and velocity (cm/sec) (B) characterizing the spontaneous locomotor activity profile of juvenile SHR rats sham operated (white columns), sham injected (grey columns), bicuculline injected (Blacked striped white columns), bicuculline injected and treated with vehicle (Blacked striped blue columns) and bicuculline injected and treated with aripiprazole (Blacked striped red columns) during adolescence. Stars indicate levels of significance (*$P<0.05$) between the antecedent and following column within each timepoint tested with Mann Whitney’s test followed by Bonferroni post hoc test.

FigS3: **Behavioral frequency changes.** Rising (A), grooming (B) and climbing (C) frequencies of juvenile bicuclline-injected rats treated with vehicle (withe columns) or aripiprazole (red columns) compared to the bicuculline-injected only (grey columns) during adolescence. No significant differences are shown between groups when tested with Mann Whitney’s test followed by Bonferroni post hoc test.

Fig.S4: **Behavioral duration and latency changes.** Rising (A), grooming (B) and climbing (C) latency and total duration of juvenile bicuclline-injected rats treated with vehicle (withe columns) or aripiprazole (red columns) compared to the bicuculline-injected only (grey columns) during adolescence. No significant differences are shown between groups when tested with Mann Whitney’s test followed by Bonferroni post hoc test.

Fig.S5: **Proton magnetic resonance spectroscopy changes.** Modulation of glutathione (GSH,) inositoles (Ins), total choline (tot. Cho), total creatine (tot. Cr), alanine (Ala), aspartate (Asp), taurine (Tau) and total total N-acetylaspartate (tot. NAA) as detected by in vivo 1H NMR MRS in the right striatum of sham operated (grey columns), sham injected (blue columns), bicuculline injected (pink columns), bicuculline injected and aripiprazole treated (purple columns) and the corresponding contralateral side (white filled columns). Ipsilateral (*) and contralateral ($^{\#}$) different significant levels are indicated for each time point (E: early, M: middle and L: late) of adolescence: *p<0.05, **p<0.01, ***p<0.001 and ****p<0.0001.

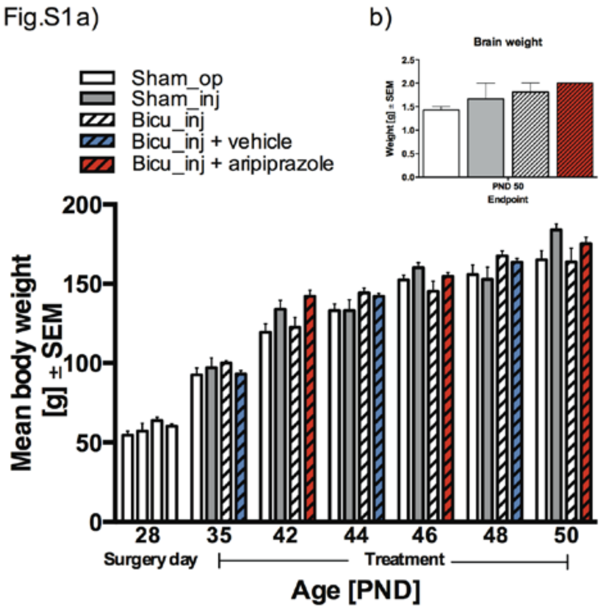

Spontaneous locomotion

Fig.S2

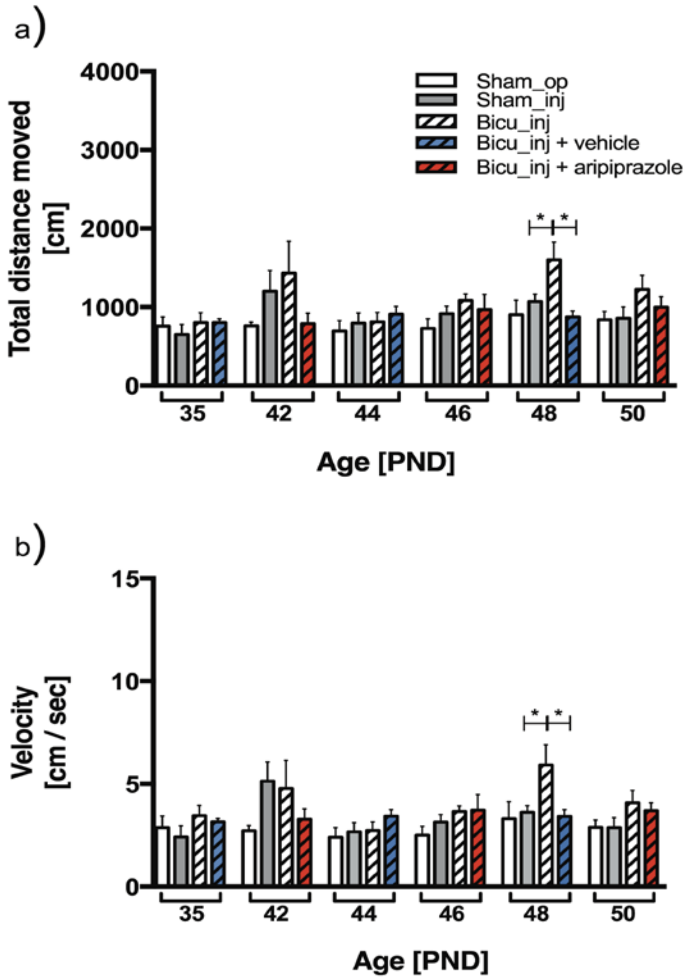

Stereotypies quantification

- Rising, grooming and climbing frequency

Fig.S3

a) b) c)

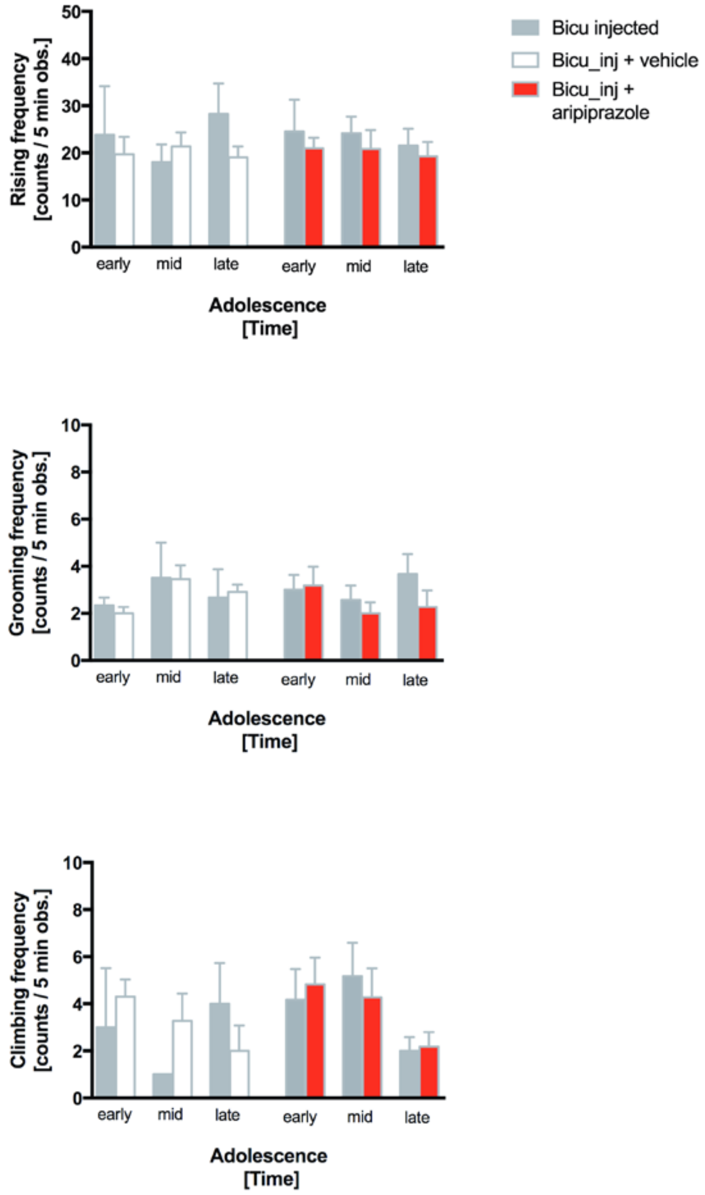

- *Rising, grooming and climbing frequency*

Fig.S4

a)

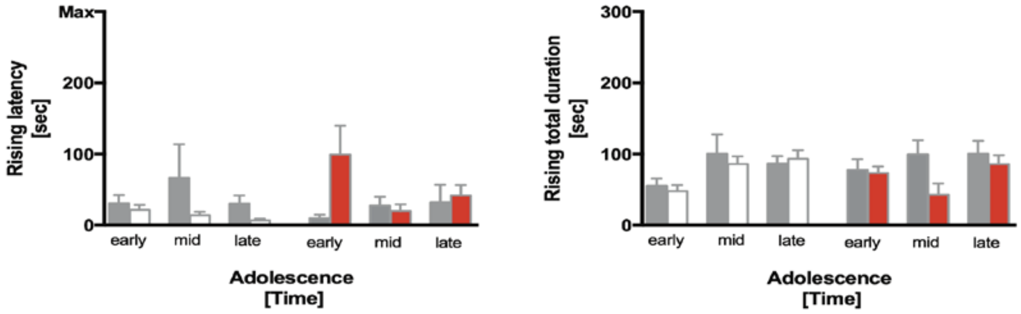

b)

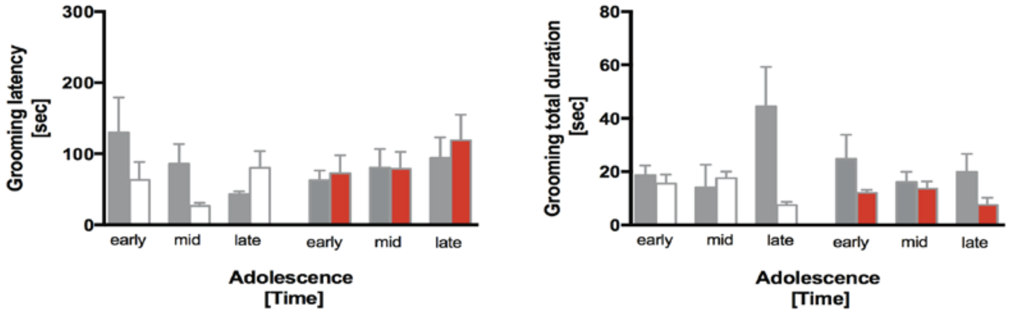

c)

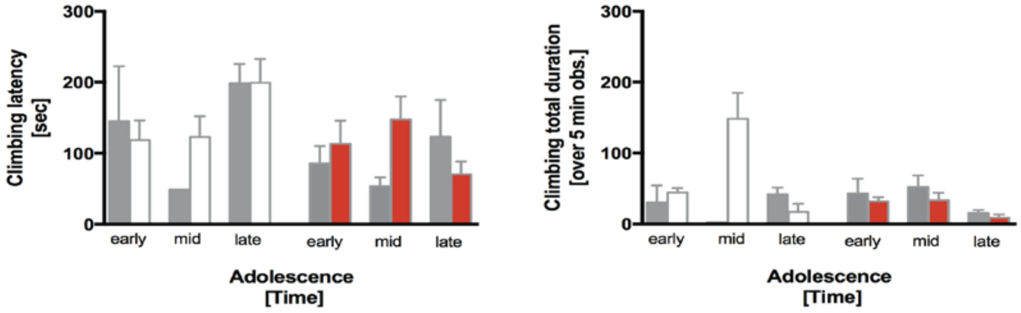

Fig.S5

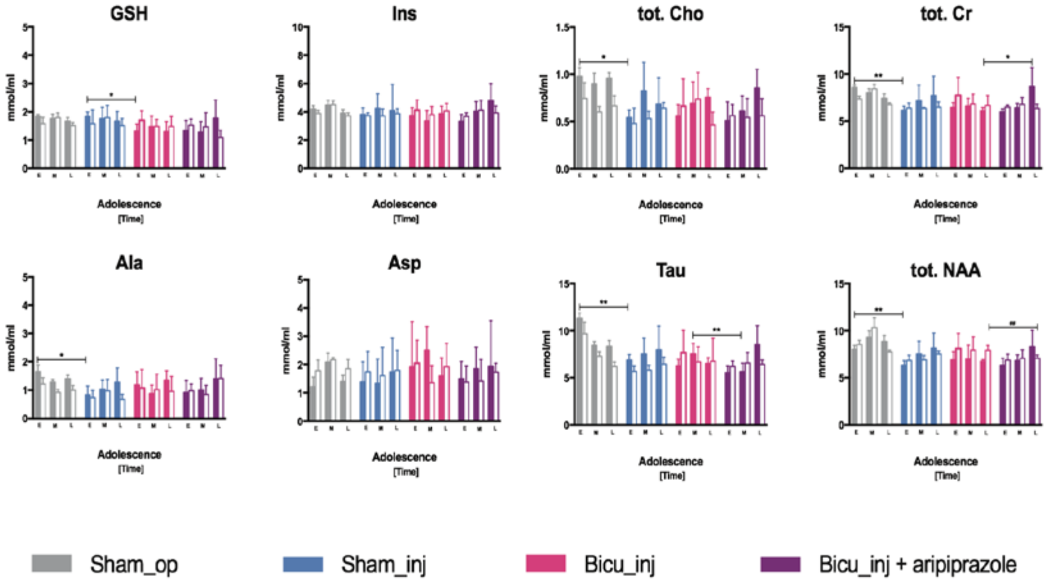
GSH
Ins
tot. Cho
tot. Cr
Ala
Asp
Tau
tot. NAA
mmol/ml
Adolescence
[Time]
Sham_op
Sham_inj
Bicu_inj
Bicu_inj + aripiprazole
