# Supplementary figures and images for: Aripiprazole Selectively Reduces Motor Tics in a Young Animal Model for Tourette’s Syndrome and Comorbid Attention Deficit and Hyperactivity Disorder
Source: Front Neurol. 2018 Feb 13;9:59. doi: 10.3389/fneur.2018.00059 (PMC5816975; doi:10.3389/fneur.2018.00059)

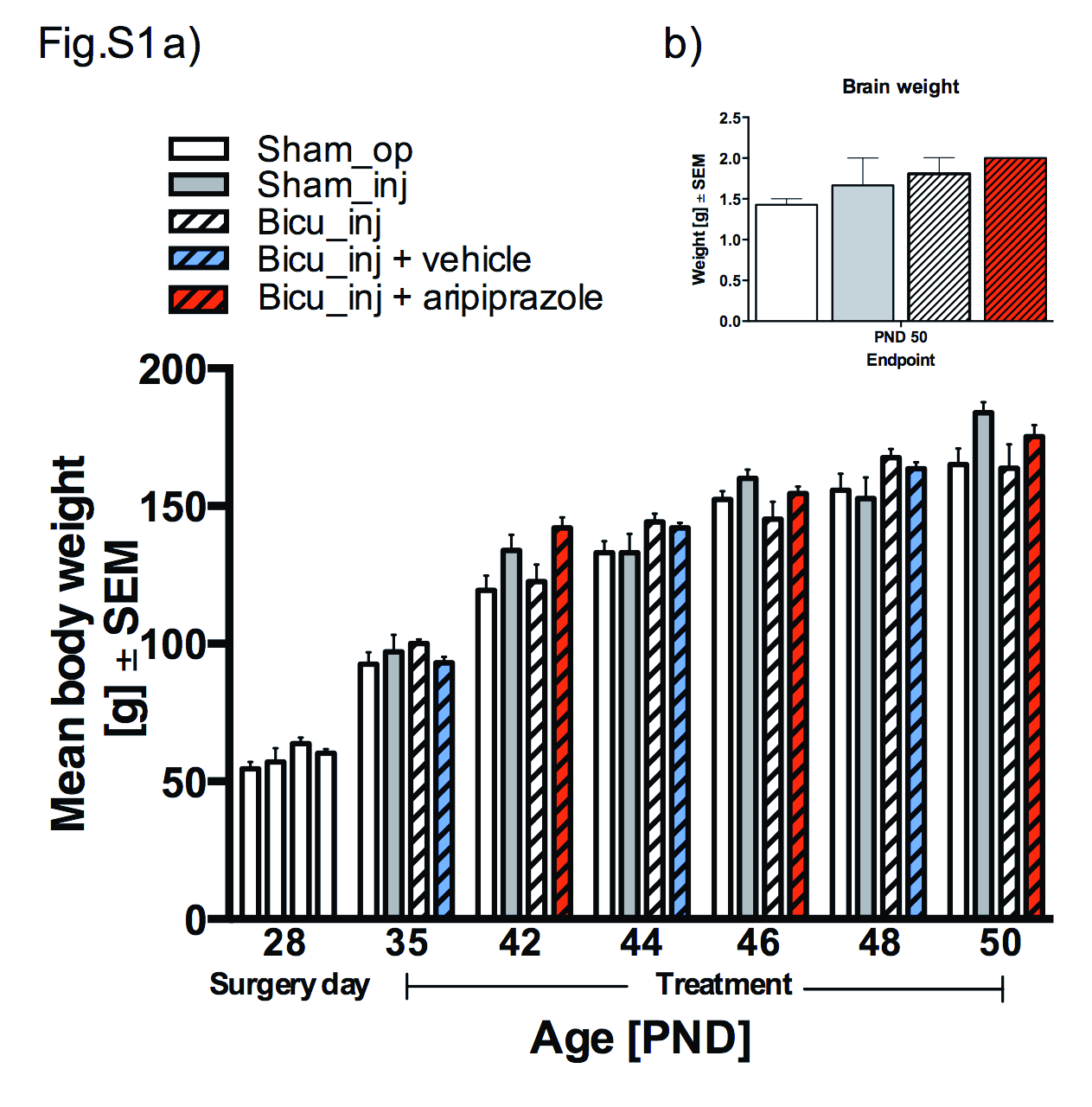

Supplement: Supplementary file 3 [file Image_1.TIFF]

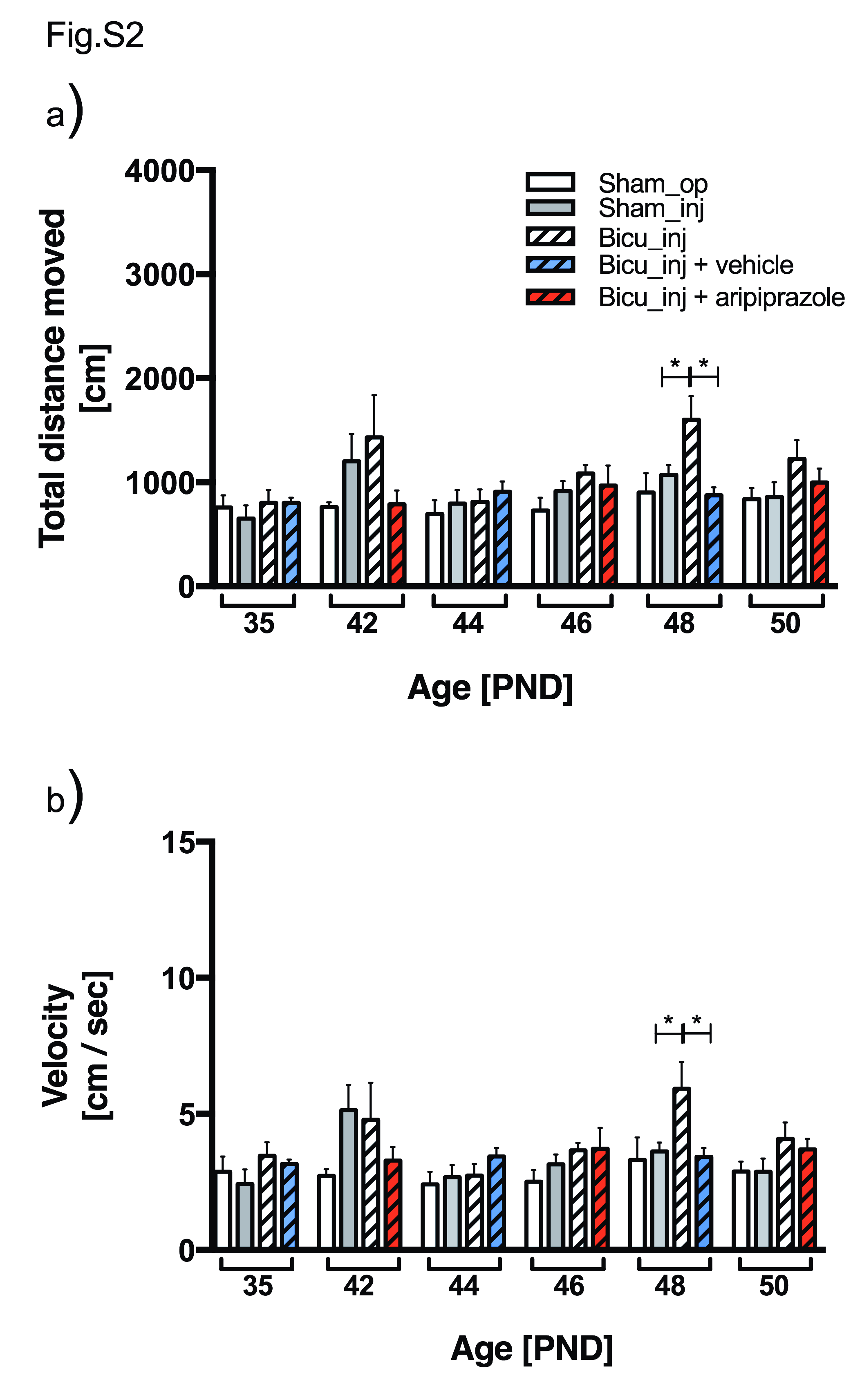

Supplement: Supplementary file 4 [file Image_2.TIFF]

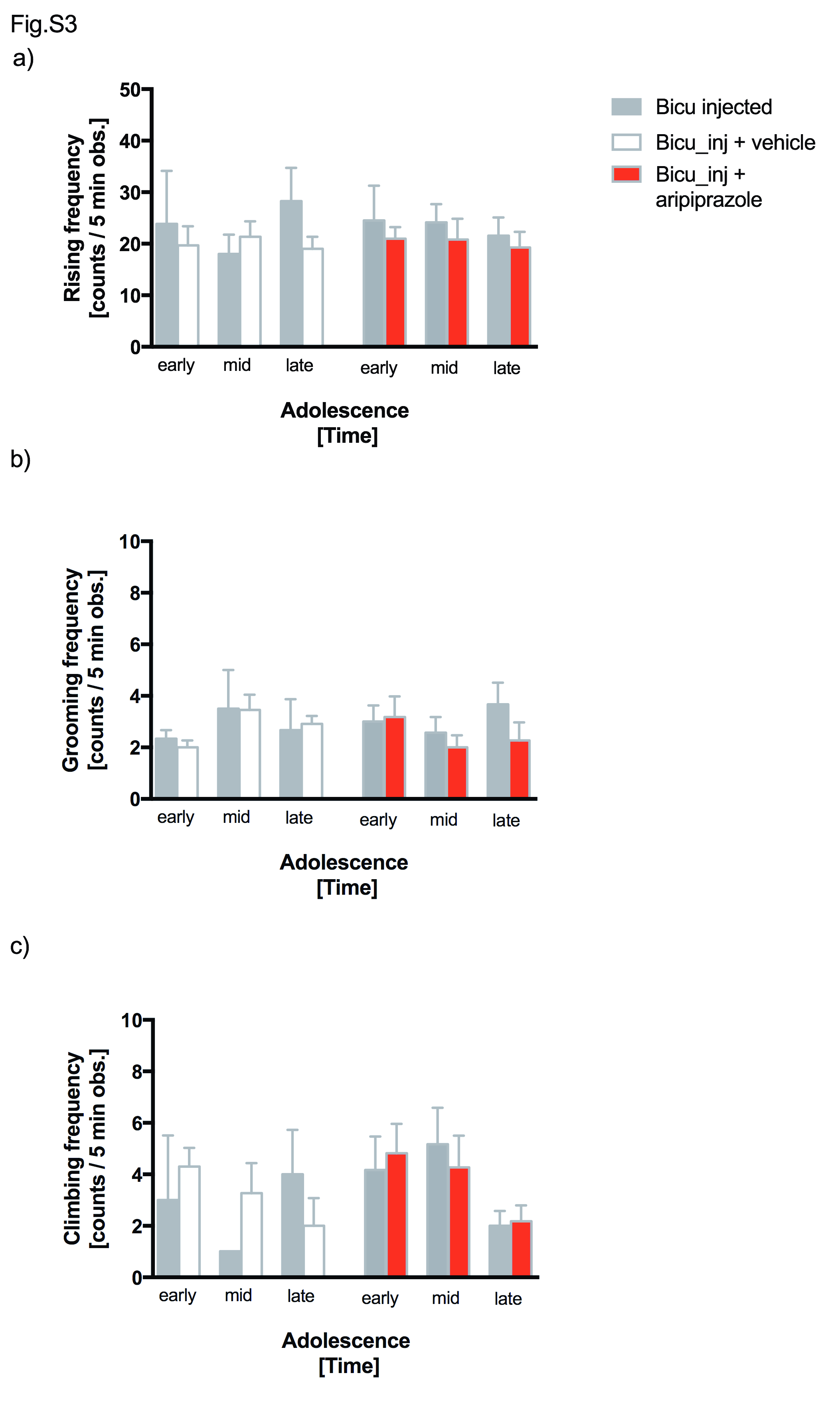

Supplement: Supplementary file 5 [file Image_3.TIFF]

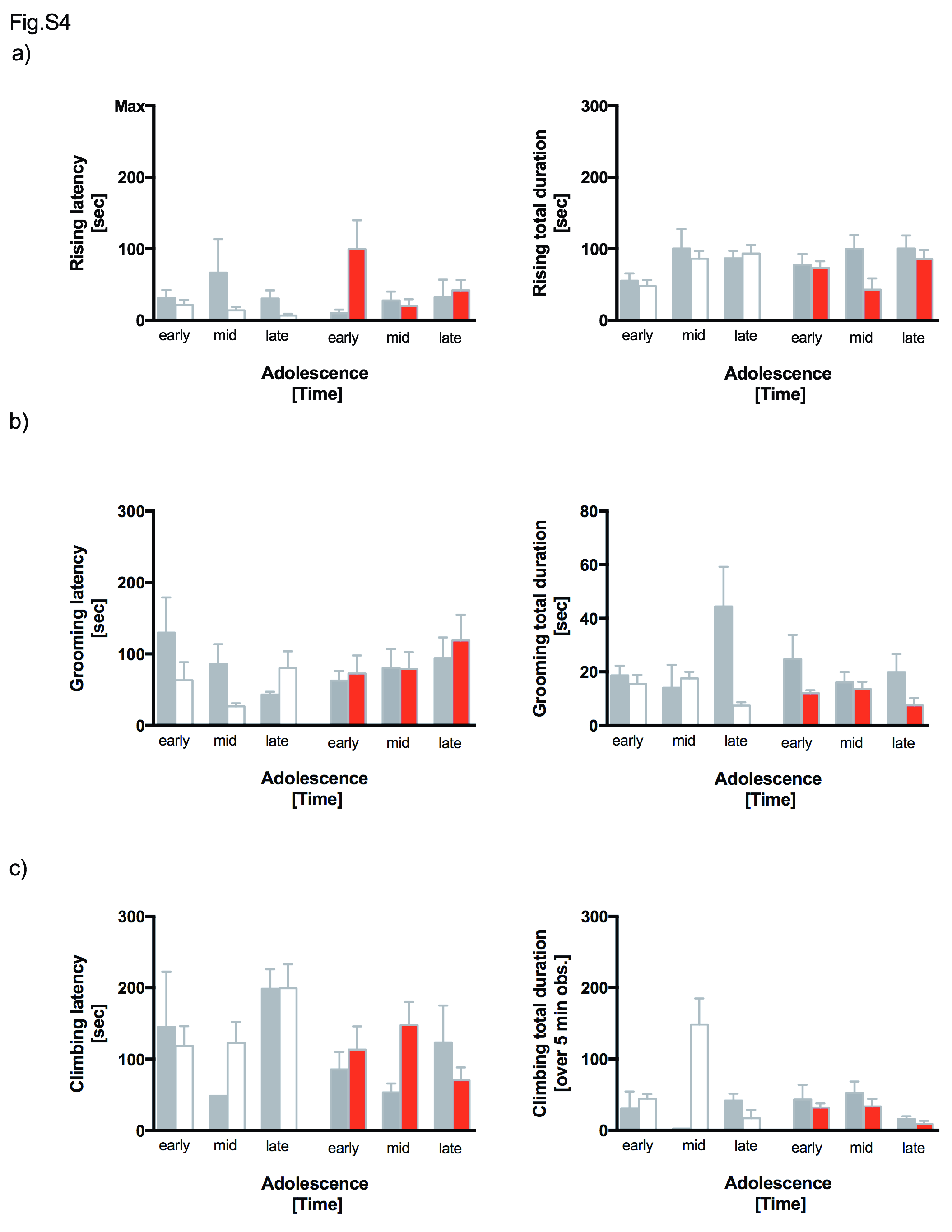

Supplement: Supplementary file 6 [file Image_4.TIFF]

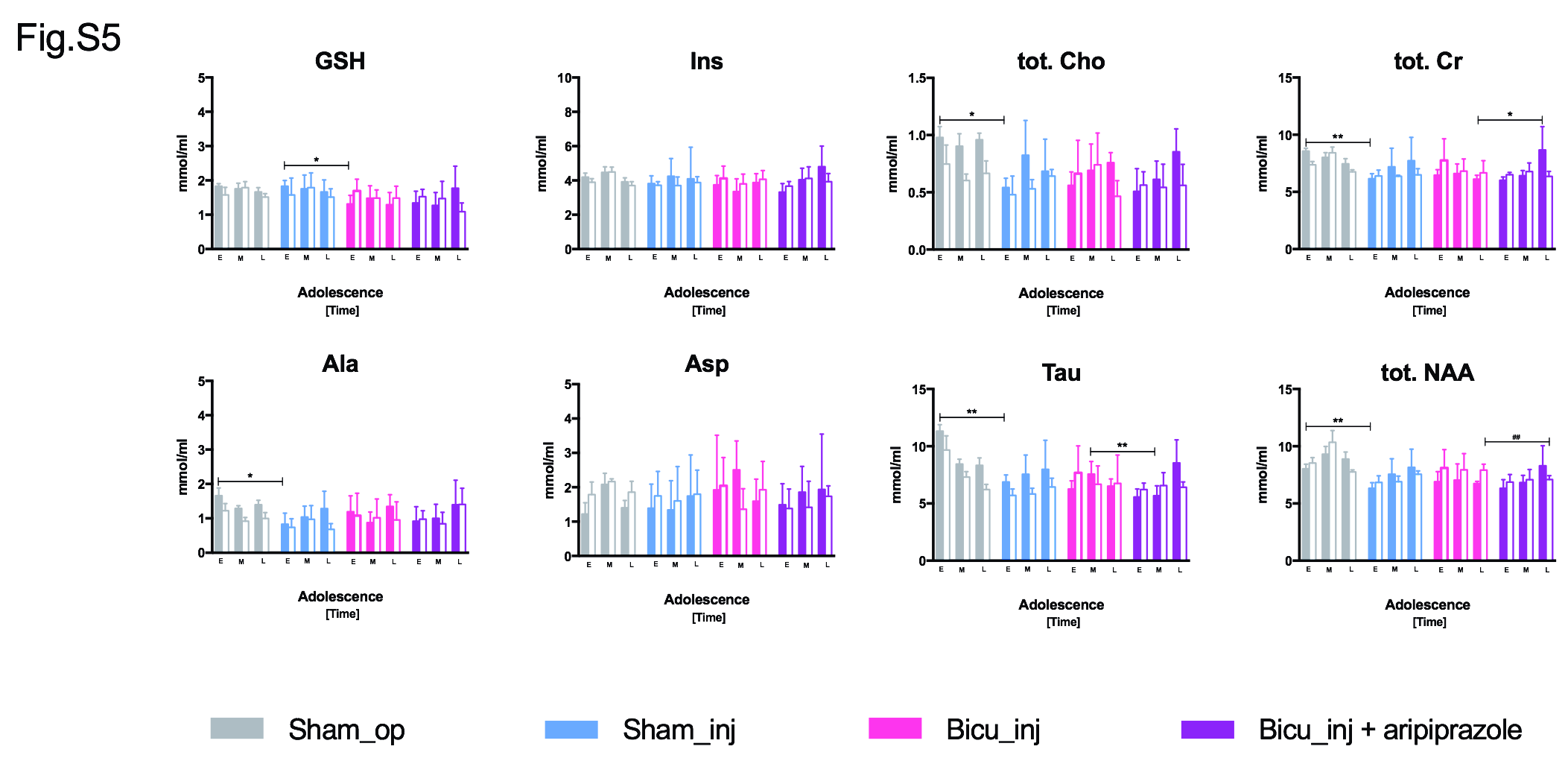

Supplement: Supplementary file 7 [file Image_5.TIFF]
